# Effect of Levodopa Initiation on the Gut Microbiota in Parkinson's Disease

**DOI:** 10.3389/fneur.2021.574529

**Published:** 2021-03-04

**Authors:** Natalia Palacios, Anas Hannoun, Julie Flahive, Doyle Ward, Kelsey Goostrey, Anindita Deb, Kara M. Smith

**Affiliations:** ^1^Department of Public Health, University of Massachusetts Lowell, Lowell, MA, United States; ^2^Department of Veterans Affairs, Edith Nourse Rogers Memorial Veteran's Hospital (ENRM VA) Hospital, Bedford, MA, United States; ^3^Department of Nutrition, Harvard T.H. Chan School of Public Health, Boston, MA, United States; ^4^Department of Neurology, Geisel School of Medicine at Dartmouth, Manchester, NH, United States; ^5^Department of Population and Quantitative Health Sciences, University of Massachusetts Medical School, Worcester, MA, United States; ^6^Department of Microbiology and Physiological Systems, University of Massachusetts Medical School, Worcester, MA, United States; ^7^Department of UMass Center for Microbiome Research, University of Massachusetts Medical School, Worcester, MA, United States; ^8^Department of Neurology, University of Massachusetts Medical School, Worcester, MA, United States

**Keywords:** Levodopa, microbiome, motor function, Parkinson's disease, Unified Parkinson's Disease Rating Scale

## Abstract

**Background:** The impact of Levodopa on the gut microbiota of Parkinson's disease (PD) patients has not been sufficiently addressed.

**Methods:** We conducted a longitudinal study to examine the impact of Levodopa initiation on the gut microbiota composition of 19 PD patients who had not previously been exposed to Levodopa. Patients provided two stool samples prior to and two samples 90 days after starting Levodopa. Motor impairment (MDS-UPDRS Part III), diet, and other patient characteristics were assessed. 16S rRNA gene amplicon sequencing was used to characterize the microbiota. We examined, cross-sectionally and longitudinally, the associations between Levodopa use and alpha and beta diversity and performed feature-wise, multivariate modeling to identify taxa associated longitudinally with Levodopa use and with improvement in motor function after Levodopa administration.

**Results:** We did not observe significant differences in alpha or beta diversity before vs. after initiation of Levodopa. In longitudinal feature-wise analyses, at the genus level, no taxa were significantly associated with Levodopa use after false discovery rate (FDR) correction (*q* < 0.05). We observed a marginally lower relative abundance of bacteria belonging to *Clostridium* group IV in PD patients who experienced a medium or large improvement in motor impairment in response to Levodopa compared to those with a small response [β = −0.64 (SE: 0.18), p-trend: 0.00015 p-FDR: 0.019].

**Conclusions:** In this study, Levodopa was not associated with changes in microbiota composition in this longitudinal analysis. The association between abundance of *Clostridium* group IV and short-term motor symptom response to Levodopa is preliminary and should be investigated in larger, longer-term studies, that include a control group.

## Introduction

Parkinson's disease (PD) is a progressive neurodegenerative disorder characterized by aggregation of the protein alpha-synuclein in Lewy bodies and neurites, leading to dopaminergic neuron loss in the substantia nigra (1). PD is typically associated with motor symptoms such as bradykinesia, tremor and rigidity, but the importance of non-motor aspects, particularly gastrointestinal problems, is increasingly appreciated. Constipation is a well-recognized risk factor for PD (2), and increased intestinal permeability and inflammation have been described in PD (3). Due to the growing evidence for gastrointestinal involvement in PD, as well as support for the hypothesis that alpha-synuclein may propagate from the gut, along the vagus nerve to the brain (4, 5), the gut microbiome holds promise as a source of biomarkers as well as for potential therapeutic intervention in PD.

To date, a number of studies have reported differences in the gut microbiota composition of PD patients compared to controls (6–18). There has also been significant heterogeneity of results. Only a single study considering disease progression has far been conducted, reporting that low counts of *Bifidobacterium* may be associated with faster PD progression over 2 years (19).

Medications have been shown to explain a large proportion of variance in the gut microbiota composition (20) and the microbiome has been suggested to impact medication efficacy (21–23). Specific to PD, the microbiome may explain the heterogeneity in the efficacy and side effects of Levodopa, which is not clearly linked with clinical factors (17). A recent report identified *E. faecalis* as potentially responsible for Levodopa decarboxylation, and potentially, the differential Levodopa response among PD patients (24). It has been shown that bacteria in the rat small intestine express genes encoding for the enzyme tyrosine decarboxylase (TDC) that decarboxylates Levodopa to dopamine, potentially suggesting a role for the microbiome in the pharmacokinetics and effect of Levodopa in individuals with PD (25).

Recent studies have suggested that some of the reported differences in gut microbiota composition of PD patients compared to controls could potentially be related to Levodopa use. In a small study that compared drug naïve (*n* = 12) and treated (*n* = 26) PD patients, Levodopa use was significantly associated with the abundances of *Bacillaceae* (9). In another study, Levodopa dose was inversely associated with abundance of genera *Dorea* and *Phascolarctobacterium* (18). A decrease in genus *Faecalibacterium* was reported in a cross-sectional analysis of microbiota composition of PD patients using Levodopa (26). Notably, in a recent study comparing the microbiota composition of PD patients vs. controls (27), adjustment for Levodopa use, in addition to other covariates, resulted in attenuation of most findings, highlighting the importance of considering medication use in analyses (27).

Understanding the impact of Levodopa on the gut microbiome is crucial to separate disease-related from medication-related impacts on the microbiome. However, to our knowledge, no study to date has examined the impact on the microbiome of starting Levodopa longitudinally in *de novo* PD. In this study, we evaluated the gut microbiota composition *de novo* PD patients prospectively and longitudinally, before and after starting Levodopa therapy. We also evaluated associations between the microbiota composition and Levodopa response in PD patients.

## Methods

### Enrollment and Study Participants

**Figure 1:**
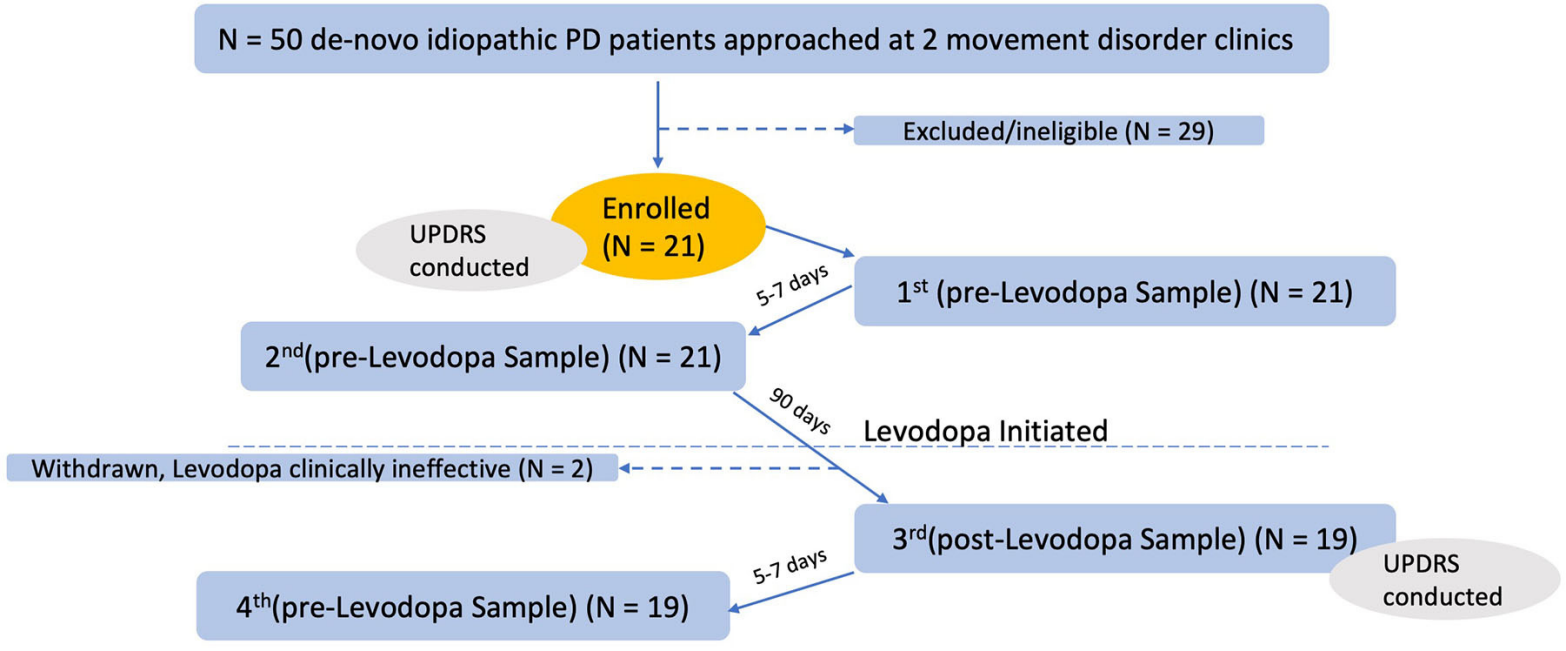
Participant enrollment, testing, and collections.

Fifty patients with idiopathic PD were approached for enrollment, and 21 were enrolled (Figure 1). Patients did not qualify for the study, if they were already on or have previously taken any Levodopa or Levodopa equivalent brand name treatment prior to the study such as Sinemet, Sinemet CR, Rytary, or Duopa gel infusion. Participants were allowed to be stable on other PD medications during the study (dopamine agonists, mono-amine oxidase inhibitors, and NMDA antagonists).

We excluded those on antibiotics, antifungals, antivirals, or antiparasites, cytokines, any immunosuppressants or immunomodulators, large or FDA approved doses of probiotics in any form, at the time of recruitment or within 6 months of stopping, as well as those who had a recent gastrointestinal inflammatory condition or major gastrointestinal surgery. Other exclusion criteria included unstable vital signs upon enrollment, acute infectious disease at the time of the sample obtaining, unstable dietary history, recent history of chronic alcohol consumption, positive HIV, HBV, or HCV infection, confirmed state or condition of immunodeficiency, pregnancy or lactation.

All study participants were enrolled through the movement disorders clinic at the University of Massachusetts Medical School and the movement disorders clinic at Brown University. Idiopathic PD diagnosis was made by a movement disorders specialist based on their best clinical judgment following the UK Brain Bank Criteria (28). After appropriate evaluation and discussion with the patient, a clinical decision to start Levodopa was made.

MDS-Unified Parkinson's Disease Rating Scale (MDS-UPDRS) was captured both upon enrollment and 90 days after starting Levodopa. Both body mass index and a dietary questionnaire were collected at enrollment and study completion. Upon enrollment, other demographic and clinical parameters were collected including Hoehn and Yahr (H&Y) Stage (29), PD duration, comorbidities, medications, and alcohol or smoking history.

### Stool Samples Collection and Storage

Samples were collected in OMNIgene∙GUT collection tubes (OMR-200, DNAgenotek) and stored at −80°C until extraction. Each patient provided a total of four samples: two samples were collected directly prior to starting Levodopa, 5–7 days apart. Ninety days after starting Levodopa, the third and fourth samples were collected, an average of 7 days apart.

### DNA Extraction and 16S rDNA Sequencing

Approximately 400 μl stool aliquots were extracted using the PowerMag® Soil DNA Isolation Kit (27100-4-EP, MO Bio Laboratories, Inc.) on an epMotion® 5075 TMX Liquid Handling Workstation. The 16S rRNA gene was sequenced following methods previously described (30) using the 341F and 806R universal primers to amplify the V3–V4 region. Three hundred nt paired-end sequences were generated on the Illumina MiSeq platform. Reads were assembled and clustered, and an OTU table was generated using the UPARSE pipeline (31). Taxonomic classifications were determined using SINTAX (31) and RDP training set v16 (with species names) (https://drive5.com/usearch/manual/sintax_downloads.html).

### Statistical Analysis

All statistical analyses were performed in R version 3.6.0 and SAS version 9.4 (SAS Institute Inc., Cary, NC). We used false discovery rate (FDR) for multiple comparisons correction.

### Omnibus Testing

We used the Vegan package in R to calculate the Shannon Alpha Diversity index. Faith's Phylogenetic diversity was calculated in QIIME2 (32). In descriptive analyses, we used the first pre-Levodopa sample from all patients to characterize the associations between microbial alpha diversity, using one way Analysis of Variance (ANOVA), and the covariates assessed in the study, including age, BMI, and diet prior to starting Levodopa. These comparisons were made for alpha diversity and Permutational Analysis of Variance (PERMANOVA) for beta diversity metrics. Subsequently, we performed a longitudinal analysis with linear mixed models, treating subject as a random effect, to examine the association between sample status (post- vs. pre-Levodopa) and the two Alpha diversity metrics (Shannon and Faith's Phylogenetic) adjusting for age, sex and BMI in all four samples from all 19 patients.

#### Beta Diversity

Beta diversity comparisons were performed using four metrics: Bray-Curtis dissimilarity, Jaccard similarity, weighted UniFrac, and unweighted UniFrac. First, to examine which predictors contribute to beta diversity before initiation of Levodopa, we used the first pre-Levodopa sample and conducted a set of univariate PERMANOVA analyses to examine whether any of the covariates contributed strongly to beta diversity, using the “adonis” command in R with 999 permutations. Subsequently, to examine whether initiation of Levodopa impacts beta diversity, we computed the dissimilarity, using the four metrics above, between the first two (pre-Levodopa) samples and compared this dissimilarity to that between the second and third (pre-to-post) and the third and fourth (post-post) samples, using the Wilcoxon signed rank test. Finally, we performed a longitudinal PERMANOVA, using all four samples, treating subject as a random effect and examining the association between Levodopa use and each of the beta diversity metrics, adjusted for age, sex and BMI.

### Feature-Wise Analyses

We used MaAsLin2 (Multivariate Association with Linear Models 2 (Version 1.4.0): https://huttenhower.sph.harvard.edu/maaslin2), a modified general linear model for feature-wise multivariate modeling, to identify differentially abundant taxa associated with Levodopa use. The MaAsLin2 model was fit with data from all four time points, treating subject as a random effect, and adjusting for age, sex, and BMI. We used the default parameters in MaAsLin2 (https://github.com/biobakery/Maaslin2).

#### Longitudinal Analyses to Identify Taxa Associated With Motor Response to Levodopa Treatment

Pre- and post-Levodopa MDS-UPDRS Part III (motor examination) was available for 16 patients. We stratified patients according to three categories of response to Levodopa, using the change in MDS-UPDRS III at 90 days compared with baseline. A small response was defined as no or minimal change: a decrease in MDS-UPDRS III or an increase <1 point. A medium response was defined as MDS-UPDRS III improvement of 1 to <12 points, and large response was defined as an improvement of difference of 12–35 points. These cut-points were based on visual inspection of MDS-UPDRS III data in order to create categories with relatively balanced numbers of patients (*n* = 5, 5, and 6 per group, respectively).

We used MaAsLin2 on all four longitudinal samples with random effects for subject to identify taxa associated with change in MDS-UPDRS III after Levodopa treatment. These analyses were conducted using change in UPDRS as a categorical (small/medium/large) outcome, and p-trend was calculated across categories.

#### Sensitivity Analyses

While the mean disease duration among our study participants was 3 years, one patient had PD for 17 years. We thus conducted sensitivity analyses excluding this participant.

### Ethics

All study procedures, documents, and advertisement methods were performed according to the rules and regulations of the Institutional Review Board (IRB) of the University of Massachusetts Medical School (Docket #H00013990). HIPAA authorization for review of medical charts for research purposes were signed by all participants.

## Results

Twenty-one patients successfully provided the first two stool samples, prior to starting Levodopa. Two patients stopped Levodopa use because it was clinically ineffective and were thus withdrawn from our study. The remaining 19 patients returned the two post-Levodopa samples (Figure 1), for a total of four stool samples per patient (two pre-Levodopa and two post-Levodopa). Demographic, clinical characteristics, and Levodopa daily dose (LDD) of the patients under study are shown in Table 1. The Levodopa daily dose at 90 days was available for 17 of the 19 patients. The majority (11 of 17) participants were taking 300 mg Levodopa per day. Other doses were as follows: 900 mg (*n* = 1), 600 mg (*n* = 2), 450 mg (*n* = 2), and 200 mg (*n* = 1). As shown in Table 1, 8 of 19 participants were stable on non-Levodopa PD medications during the study (dopamine agonists, mono-amine oxidase inhibitors, and NMDA antagonists). Participants did not initiate any other PD medication during the study.

**Table 1:** Demographic characteristics of study participants.

Age [years, mean (SD)]	71 (5.3)
Sex [*n* (%) female]	9 (47)
Race [*n* (%) white]	100
Ethnicity [*n* (%) non-Hispanic]	100
BMI [mean (SD)]	28 (5.8)
PD duration (years) [mean (SD)]^a^	3 (3.7)
Smoking [*n* (%)]	0
Alcohol (drinks/week) [mean (SD)]	1.6 (2.9)
Comorbidities [*n* (%)]	
Hypertension	7 (37)
Hyperlipidemia	9 (47)
Diabetes	3 (16)
Medications [*n* (%)]	
Dopaminergic medications	8 (42)
Metformin	2 (11)
Statins	7 (37)
Antidepressants (SSRIa and SNRIs)	3 (16)
MDS-UPDRS Part III-pre [mean (SD)]	39 (18)
MDS-UPDRS Part III-post [mean (SD)]	29 (19)
MDS-UPDRS Part III-difference [mean (SD)]	10 (12)
Levodopa daily dose [mean (range)]	382.9 (200–900)

a *One participant had PD duration of 17 years*.

### Alpha Diversity

**Figure 2:**
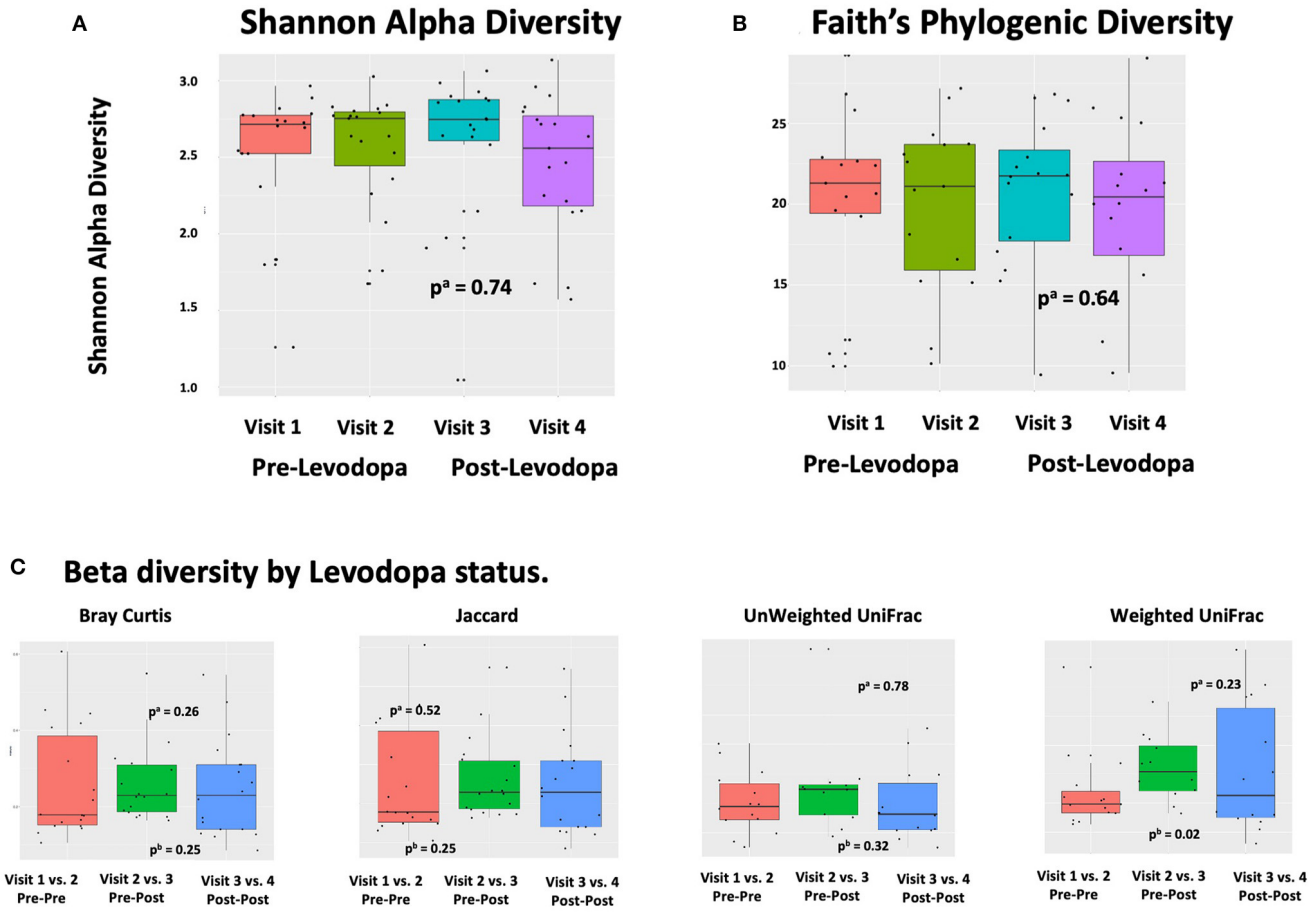
Alpha and Beta Diversity of the gut microbiome in relation to Levodopa use. **(A,B)** Alpha diversity metrics (Shannon Alpha Diversity and Faith's Phylogenic Diversity) in relation to sample and timing of Levodopa use. *P*-values were calculated based on ^a^linear mixed model (LMM) with alpha diversity (Shannon or Faith's Phylogenetic Diversity) as the outcome and Levodopa status (pre vs. post) as predictor, adjusted for gender, age, PD duration, and BMI, treating subject as a random effect. “Visit” refers to study visit, where visits 1 and 2 took place prior to Levodopa administration and visits 3 and 4 took place after. **(C)** Beta diversity metrics (Bray Curtis, Jaccard, unweighted UniFrac, weighted UniFrac) in relation to sample and timing of Levodopa use. Comparisons are made for each of Bray Curtis, Jaccard, unweighted Unifrac, and weighted Unifrac, comparing the dissimilarity between sets of samples from visit 1 vs. visit 2 (both pre-Levodopa samples), to dissimilarity between visit 2 vs. visit 3 (spanning initiation of Levodopa), and to that between visit 3 vs. visit 4 (both post-Levodopa samples). *P*-values were computed with ^a^longitudinal PERMANOVA incorporating all four longitudinal samples, adjusting for age, BMI, and treating subject as a random effect and ^b^Wilcoxon rank sum test comparing Pre-Post (dissimilarities of samples 2, 3) and Pre-Pre groups (dissimilarities of samples 1, 2) groups.

In linear regression analyses in pre-Levodopa samples, Shannon alpha diversity was positively associated with age (β = 0.05; *p* = 0.005) and marginally inversely associated with BMI (β = 0.03, *p* = 0.07). Faith's Phylogenetic diversity was positively associated with PD duration (β = 11.16, *p* = 0.05) as well-alcohol use (β = 0.91, *p* = 0.04), and inversely associated with BMI (β = −0.23, *p* = 0.04), and marginally inversely associated with age (β = 0.49, *p* = 0.09) (Table 2). In longitudinal linear mixed model analyses, examining the association between Levodopa status (sample taken before Levodopa vs. after Levodopa) after adjustment for gender, age, PD duration, BMI, treating subject as a random effect, and alpha diversity metrics, we did not observe significant associations between Levodopa status and Shannon Alpha Diversity (β = −0.02, *p* = 0.74) or Faith's (β = −0.21, *p* = 0.64) (Figures 2A,B). Excluding the participant with longer (17 y) disease duration did not substantially impact the alpha diversity results.

**Table 2:** Associations between alpha and beta diversity and study covariates at baseline.

	**Alpha diversity^a^** **(*****p*****-value)**		**Beta diversity^b^** ***R***^****2****^ **(*****p*****-value)**			
	**Shannon**	**Faith's**	**Bray-Curtis**	**Jaccard**	**Weighted UniFrac**	**Unweighted UniFrac**
Hoehn and Yahr stage (per 1 unit)	0.06 (0.60)	0.99 (0.52)	0.046 (0.63)	0.09 (0.10)	0.05 (0.50)	0.05 (0.53)
Age (per 1 year)	**0.05 (0.02)**	0.49 (0.09)	0.10 (0.09)	0.07 (0.24)	0.09 (0.14)	0.09 (0.15)
BMI (per 1 unit)	−0.03 (0.07)	−0.23 (0.04)	**0.12 (0.047)**	0.06 (0.45)	0.08 (0.20)	0.08 (0.22)
PD duration (pear 1 year)	0.26 (0.47)	**11.16 (0.05)**	0.10 (0.62)	**0.30 (0.004)**	0.10 (0.64)	0.10 (0.62)
Sex (F vs. M)	0.12 (0.58)	3.11 (0.31)	0.030 (0.91)	0.04 (0.81)	0.05 (0.53)	0.05 (0.53)
Dopaminergic medication use^c^	0.006 (0.97)	−1.79 (0.58)	0.046 (0.59)	0.07 (0.24)	0.01 (0.99)	0.01 (0.99)
Antidepressants^c^	0.27 (0.36)	−0.62 (0.89)	0.046 (0.61)	0.06 (0.35)	0.08 (0.18)	0.08 (0.19)
Statins^c^	0.06 (0.79)	1.25 (0.69)	0.055 (0.45)	0.06 (0.43)	0.05 (0.64)	0.05 (0.68)
Metformin^c^	−0.31 (0.37)	−4.41 (0.32)	0.077 (0.19)	0.04 (0.83)	0.08 (0.23)	0.08 (0.24)
Diabetes^c^	−0.44 (0.13)	−1.79 (0.64)	0.039 (0.75)	0.06 (0.36)	0.05 (0.63)	0.05 (0.63)
Yogurt^c^	0.30 (0.16)	3.33 (0.28)	0.046 (0.62)	0.06 (0.35)	0.05 (0.65)	0.05 (0.63)
Whole grains daily^c^	0.01 (0.95)	1.79 (0.58)	0.05 (0.48)	0.06 (0.37)	0.05 (0.58)	0.05 (0.58)
Meat daily^c^	−0.03 (0.90)	−3.21 (0.29)	0.06 (0.42)	0.24 (0.07)	0.06 (0.43)	0.06 (0.44)
Nuts daily^c^	0.33 (0.15)	2.43 (0.45)	**0.123 (0.048)**	0.05 (0.67)	0.07 (0.30)	0.07 (0.30)
Fruits and vegetables daily^c^	−0.17 (0.63)	−4.24 (0.34)	0.05 (0.57)	0.04 (0.70)	0.05 (0.53)	0.05 (0.54)
Alcohol use weekly^c^	0.05 (0.19)	**0.91 (0.04)**	0.11 (0.07)	0.06 (0.29)	0.10 (0.08)	0.11 (0.09)
Caffeinated beverages daily^c^	0.05 (0.83)	2.52 (0.42)	0.06 (0.33)	0.03 (0.98)	0.04 (0.80)	0.04 (0.81)

a *Univariate linear regression model with alpha diversity measure as the outcome*.

b *Permutational Multivariate Analysis of Variance (PERMANOVA)*.

c *Binary (yes/no) indicators. Values with p ≤ 0.05 are bolded*.

### Beta Diversity

In descriptive analyses within the first, pre-Levodopa sample, daily intake of nuts (*R*^2^ =0.12, *p* = 0.047), BMI (*R*^2^ = 0.12, *p* = 0.040), alcohol use (*p* = 0.07, *R*^2^ = 0.11), and age (*p* = 0.09, *R*^2^ = 0.10) explained the most variance in terms of Bray Curtis dissimilarity in univariate PERMANOVA analyses. Likewise, when considering Jaccard distances, PD duration (*p* = 0.004, *R*^2^ = 0.30) and daily meat consumption (*p* = 0.07, *R*^2^ = 0.24) explained the most variance. When considering weighted and unweighted UniFrac, none of the metadata were significantly associated with beta diversity.

In longitudinal analyses, weighted UniFrac distance was significantly greater when comparing the 2nd vs. 3rd samples (when Levodopa was initiated) then it was comparing the samples collected during the 1st vs. 2nd visits (pre-Levodopa), indicating an increase in weighted UniFrac associated with Levodopa initiation (*p* = 0.02 using Wilcoxon rank sum test comparing Pre-Post and Pre-Pre groups). Bray Curtis dissimilarities, Jaccard or unweighted Unifrac comparing the first to second (both pre-Levodopa), second to third (pre- vs. post-Levodopa), or the third to fourth (both post-Levodopa) samples were not significantly different. In longitudinal PERMANOVA analysis incorporating all four longitudinal samples, treating subject as a random effect and adjusting for age, sex, and BMI as fixed effects, Levodopa use was not associated with any of the dissimilarity metrics (Figure 2C).

### Feature-Wise Analyses

In longitudinal feature-wise analyses in MaAsLin2 adjusted for age, sex and BMI, treating subject as a random effect, no taxa were significantly associated with Levodopa use after FDR correction (*q* < 0.05). Excluding the participant with longer (17 y) disease duration did not substantially change the association between Levodopa initiation and weighted UniFrac distance (*p* = 0.04 using Wilcoxon rank sum test comparing Pre-Post (2nd vs. 3rd) and Pre-Pre (1st vs. 2nd visit) groups). The results of other analyses were also not substantially altered by the exclusion of the participant with longer PD duration.

#### The Role of Microbiota Composition in Levodopa Response

**Figure 3:**
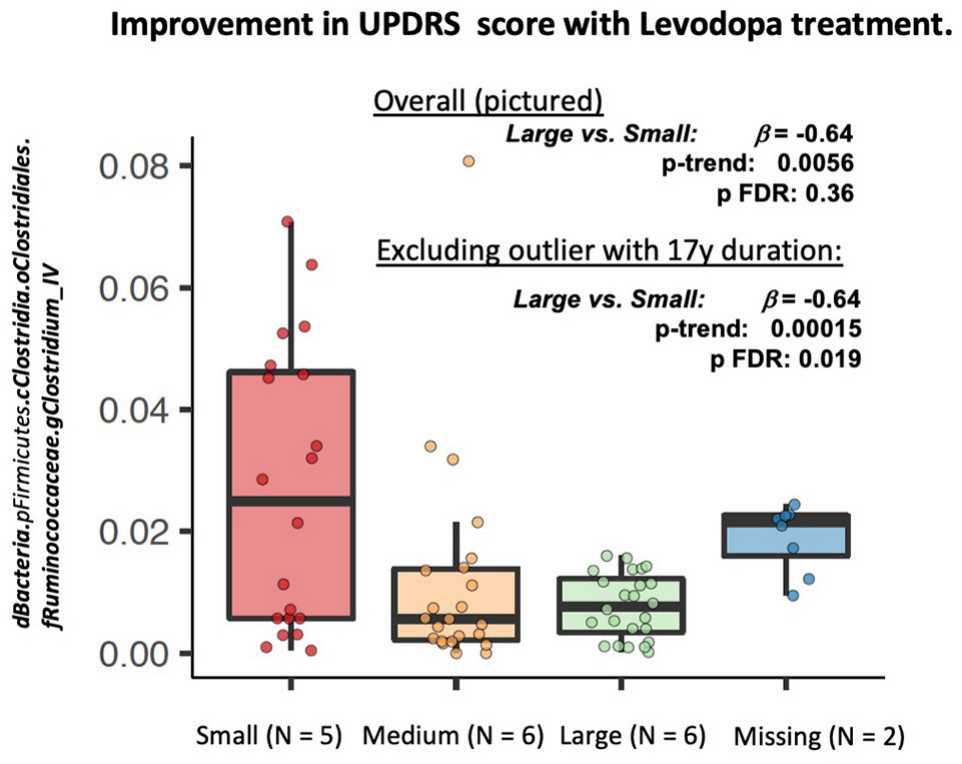
Longitudinal feature-wise analyses of change in MDS-UPDRS Part III in response to Levodopa. MaAsLin2 (Multivariate Association with Linear Models, Version 2: https://huttenhower.sph.harvard.edu/maaslin2), was fit to identify differentially abundant taxa associated with Levodopa use. The MaAsLin2 model was fit with data from all four time points, treating subject as a random effect, and adjusting for age, sex, and BMI. We used the default parameters in MaAsLin2 (https://github.com/biobakery/Maaslin2) .

In longitudinal PERMANOVA analysis, permuting within patient, Levodopa status and time, and adjusting for age and BMI, change in MDS-UPDRS score use was not associated with Bray Curtis dissimilarity (*R*^2^ = 0.093, *p* = 0.84). In longitudinal feature-wise analyses in MaAsLin2, adjusted for age, sex, and BMI, we observed a marginally lower abundance of bacteria belonging to the genus *Clostridium* group IV in patients who experienced a large response to Levodopa compared to those with the smallest response [β = −0.64 (SE: 0.18), p-trend: 0.0056, p-FDR: 0.36] This association was strengthened when excluding the participant with 17 y disease duration [β = −0.64 (SE: 0.18), p-trend: 0.00015, p-FDR: 0.019] (Figure 3).

## Discussion

There is growing evidence that the gut microbiome plays a role in PD (6, 8, 9, 11–16). Despite the increasing interest in this field, most of the studies conducted thus far have focused on microbiota comparisons between medicated PD patients and controls, using a cross-sectional approach (6, 8, 9, 11, 12, 14, 15). To our knowledge, no study to date has addressed, using a prospective and longitudinal design, whether initiation of Levodopa in *de novo* PD patients might alter the microbiome or whether the microbiome of PD patients might impact the improvement in motor function in response to initiation of Levodopa by PD patients.

In this longitudinal investigation of *de novo* PD patients who provided stool samples before and after initiation of Levodopa, we examined whether Levodopa administration alters the PD gut microbiota composition and whether composition of the gut microbiota in PD patients can predict response to Levodopa. In our baseline sample, age, and BMI were the factors most strongly associated with the microbiota (both alpha and beta diversity), which is in agreement with other studies (20). We observed an increase in weighted UniFrac distance, but not other metrics of alpha or beta diversity, when comparing the 2nd vs. 3rd samples, between which Levodopa was initiated, with the distance between 1st vs. 2nd samples (both pre-Levodopa). This suggests that Levodopa initiation is potentially associated with an increase in weighted UniFrac distance. Levodopa initiation was not associated with short-term taxonomic changes in longitudinal analysis. Our finding that only Weighted UniFrac distances, but not other metrics of beta diversity were associated with Levodopa initiation, is not necessarily conflicting with the null results for other metrics, but suggests that these effects may be more nuanced. Both Bray-Curtis and Jaccard consider discrete taxonomic groups. The limitation of Bray-Curtis and Jaccard is that taxonomic groups are equally weighted even though the phylogenetic relationships between taxonomic groups are not equal. UniFrac considers phylogenetic groups, with Unweighted UniFrac emphasizing the presence or absence of these groups and Weighted UniFrac additionally considering their abundance. Thus, Weighted UniFrac, which was the metric significantly associated with Levodopa initiation in our study, is unique among the four metrics used in that it considers the abundance of phylogenetic groups and the association we observed with this metric suggests that the abundance, more so than the presence/absence, of a phylogenetic group is contributing to statistically significant beta diversity differences.

In this study, we observed a marginally lower abundance of *Clostridium* group IV in PD patients with a good response to Levodopa. Bacteria belonging to *Clostridium* group IV are commensal, gram positive, and strict anaerobe bacteria. They play an important role in gut homeostasis and ferment dietary fiber to short chain fatty acids (SCFA), particularly butyrate, compounds that provide energy for colonocytes and play a key role in the health of the colon (33). They have also been shown to play an important role in the secondary metabolism of bile acids (34). Relevant to PD, prior work has implicated commensal *Clostridia* in the production of catecholamines, including norepinephrine and dopamine (35). *Clostridia* spp., are thought to impact host immunological signaling and immunological development, likely via production of metabolites such as SCFA and secondary bile acid metabolism (36). Specifically, *Clostridium* group IV have been shown to strongly induce of the accumulation of T regulatory cells, which are key to immune homeostasis, in the colon of mice (37). Immune system dysfunction involving neuroinflammation (38), T cell infiltration (39), and microglia activation (40) have been implicated in PD and suggested to play a role in the degeneration of dopaminergic neurons. The implication of *Clostridium* group IV in Levodopa response reported here, if confirmed in larger studies, supports the need to investigate the role of SCFA and secondary bile acids in PD. However, due to the very small sample size of this study, this result is preliminary and should be treated with caution.

Prior studies have linked Levodopa use to abundance of *Bacillaceae* (9) *Dorea* and *Phascolarctobacterium* (18). Our study did not identify any taxa significantly associated with Levodopa use but preliminarily implicated *Clostridium* group IV in Levodopa response, thus adding to an already heterogenous literature. Differences in study design size and patient population may explain some of the differences in findings from prior investigations. There may be several mechanisms by which microbiome changes can impact Levodopa response, including but not limited to impact on gastric emptying, gut mucosa local factors such as inflammation and metabolite concentrations, and direct metabolism of L-dopa via production of decarboxylases. Different mechanisms may be more important in different patients, potentially depending on patient characteristics, disease profile, and Levodopa formulation.

The longitudinal aspect of our study, and our focus on the impact of Levodopa initiation in the microbiota in *de novo* PD, is a major strength of our study. Examining microbiota composition changes within individuals longitudinally limits potential confounding due to between-person variation in diet, disease severity, and other factors that may impact studies relying on between-subject comparisons.

Our study is preliminary and was limited in power by the low number of patients, which reflected the challenge of identifying *de novo* PD patients at the time of Levodopa initiation. With adjustment for three covariates (age, sex, and BMI), with 19 total participants, each providing four samples, our study had 80% power to detect a 0.279—unit difference in common taxa (~3,792 sequences and 0.0758421 relative abundance at 100% read usage rate), and a 0.108—unit difference in rare taxa (~581 sequences and 0.0116187 relative abundance at 100% read usage rate). Most realistic microbiome effects are likely to be much smaller, and we had limited power to detect those. Larger, better-powered studies are thus needed to examine the microbiota changes after initiation of Levodopa in PD patients. Due to constrains on power, we kept our statistical models parsimonious, adjusting our analyses for age, sex, and BMI only. Several additional confounding or stratification factors could be of potential interest in future, better powered studies, including initial disease severity or disease phenotype, which could both influence extent of response to Levodopa. While this study could not take these factors into account, they would be important to consider in future analyses. Another important limitation of our study is that it did not include a healthy control group, and we were thus not able to reproduce results from prior case-control studies that made such comparisons.

Furthermore, the study focused on short-term Levodopa use and thus cannot address the question of whether longer term use Levodopa could be associated with changes in the gut microbiome. Although we did not have an untreated PD control group, it is less likely that substantial microbiota changes would have occurred, without treatment or intervention, during the 90-day study period. An additional limitation is the use of 16S sequencing, which can limit taxonomic identification. Larger, longer-term studies, with more detailed specimen analysis, such as metagenomics or multi-omic approaches are needed to validate our results and better understand the mechanism of the connections between the gut microbiome and PD pathophysiology, progression, and response to Levodopa and other PD medications.

In summary, we did not observe significant shifts in microbial alpha or beta diversity with Levodopa treatment in this short-term study of *de novo* PD patients. The lower abundance of *Clostridium* group IV in PD patients who had a good motor response to Levodopa compared to those who had no response is preliminary and should be confirmed in future studies.

## Data Availability Statement

The data that support the findings of this study are available on request from the corresponding author. The data are not publicly available due to privacy or ethical restrictions.

## Ethics Statement

The studies involving human participants were reviewed and approved by Institutional Review Board (IRB) of the University of Massachusetts Medical School (Docket #H00013990). The patients/participants provided their written informed consent to participate in this study.

## Author Contributions

AH conceived of the research project, organized and executed the project, including participant clinical and biospecimen data collection. He contributed to data analysis and interpretation, and contributed to writing the first draft and revising the manuscript. NP designed and executed statistical analyses, and reviewed and critiqued results, and contributed to writing the first draft and revising the manuscript. JF contributed to the design and execution of the statistical analysis. DW performed sample extraction, microbiome sequencing and analysis, and contributed to design and review/critique of the statistical analysis, and the revising of the manuscript. KG contributed to organization and execution of the research project, and revising of the manuscript. AD contributed to execution of the research project (participant recruitment), and review and critique of the results and the manuscript. KS contributed to study design and organization, participant recruitment and data collection, reviewed and critiqued results, and revised the manuscript. All authors contributed to the article and approved the submitted version.

## Conflict of Interest

The authors declare that the research was conducted in the absence of any commercial or financial relationships that could be construed as a potential conflict of interest.
